# Evaluation of Dietary Effects on Hepatic Lipids in High Fat and Placebo Diet Fed Rats by *In Vivo* MRS and LC-MS Techniques

**DOI:** 10.1371/journal.pone.0091436

**Published:** 2014-03-17

**Authors:** Jadegoud Yaligar, Venkatesh Gopalan, Ong Wee Kiat, Shigeki Sugii, Guanghou Shui, Buu Duyen Lam, Christiani Jeyakumar Henry, Markus R. Wenk, E. Shyong Tai, S. Sendhil Velan

**Affiliations:** 1 Laboratory of Molecular Imaging, Singapore Bioimaging Consortium, A*STAR, Singapore, Singapore; 2 Life Sciences Institute, National University of Singapore, Singapore, Singapore; 3 Department of Biochemistry and Department of Biological Sciences, National University of Singapore, Singapore, Singapore; 4 Singapore Institute for Clinical Sciences, A*STAR, Singapore, Singapore; 5 Department of Medicine, National University of Singapore, Singapore, Singapore; INRA, France

## Abstract

**Background & Aims:**

Dietary saturated fatty acids contribute to the development of fatty liver and have pathogenic link to systemic inflammation. We investigated the effects of dietary fat towards the pathogenesis of non-alcoholic fatty liver disease by longitudinal *in vivo* magnetic resonance spectroscopy (MRS) and *in vitro* liquid chromatography coupled with mass spectrometry (LC-MS).

**Methods:**

All measurements were performed on rats fed with high fat diet (HFD) and chow diet for twenty four weeks. Longitudinal MRS measurements were performed at the 12^th^, 18^th^ and 24^th^ weeks. Liver tissues were analyzed by LC-MS, histology and gene transcription studies after terminal *in vivo* experiments.

**Results:**

Liver fat content of HFD rats for all ages was significantly (*P*<0.05) higher compared to their respective chow diet fed rats. Unsaturation indices estimated from MRS and LC-MS data of chow diet fed rats were significantly higher (*P*<0.05) than HFD fed rats. The concentration of triglycerides 48∶1, 48∶2, 50∶1, 50∶2, 50∶3, 52∶1, 52∶2, 52∶3, 54∶3 and 54∶2 was significantly higher (*P*<0.05) in HFD rats. The concentration for some polyunsaturated triglycerides 54∶7, 56∶8, 56∶7, 58∶11, 58∶10, 58∶9, 58∶8 and 60∶10 was significantly higher (*P*<0.05) in chow diet fed rats compared to HFD rats. Lysophospholipid concentrations including LPC and LPE were higher in HFD rats at 24 weeks indicating the increased risk of diabetes. The expression of CD36, PPARα, SCD1, SREBF1 and UCP2 were significantly upregulated in HFD rats.

**Conclusions:**

We demonstrated the early changes in saturated and unsaturated lipid composition in fatty liver by *in vivo* MRS and *ex vivo* LC-MS. The higher LPC concentration in HFD rats indicated a higher risk of developing diabetes. Early metabolic perturbations causing changes in lipid composition can be evaluated by the unsaturation index and correlated to the non alcoholic fatty liver disease.

## Introduction

Non alcoholic fatty liver disease (NAFLD) results from an imbalance between lipid availability (from circulating lipid uptake or de novo lipogenesis) and lipid disposal (via fatty acid oxidation or triglyceride-rich lipoprotein secretion). The accumulated lipids induce oxidative stress, resulting in production of cytokines and reactive oxygen species which in turn activate apoptosis thereby initiating a sequence of disease events from steatosis to nonalcoholic steatohepatitis (NASH), which progress into fibrosis and cirrhosis [1], [2], [3], [4], [5]. Hepatic steatosis causes insulin resistance which may act as pathogenic link between obesity and its metabolic complications [6], [7]. Many patients suffering from metabolic syndrome involving obesity, dyslipidemia, hypertension, insulin-resistant type-2 diabetes mellitus and atherosclerotic cardiovascular disease are associated with NAFLD [1], [2], [3], [4], [5].

The estimation of saturated and unsaturated lipids during the transition of normal liver to NAFLD provides information on early biochemical changes and may be utilized to evaluate response during interventions including exercise and drugs. Indeed, the composition of the fatty acids (FA, the building blocks for triglyceride) in the triglycerides (TG) is suspected to contribute to the pathogenesis of NAFLD. In particular, saturated fatty acids play a prominent role in the progression of obesity and diabetes [8], [9], [10], [11]. Diets with high saturated fatty acids are associated with insulin resistance and NAFLD [12]. Saturated fatty acids are poorly oxidized compared to unsaturated fat and hence are more likely to accumulate in insulin resistant tissues [13]. Saturated lipids are also the most inhibitory lipids on insulin sensitivity [14], [15]. The degree of unsaturation of the FAs modulate the metabolic signaling and energy metabolism [16]. As such, the assessment of both the ratio of saturated and unsaturated fat in the liver, as well as the quantity of fat in the liver represent an important aspect in the pathogenesis of chronic liver disease and metabolic disease.

Magnetic resonance spectroscopy (MRS) is a non-invasive method for investigating metabolism allowing longitudinal studies on humans and rodents. It estimates both the total fat content and unsaturation index. However, its main limitation deals with the impossibility of estimating the individual lipid composition. On another hand, liquid chromatography with mass spectrometry (LC-MS) permits analysis of individual saturated and unsaturated lipids.

In this study, we used *in vivo* MRS to evaluate the longitudinal changes in liver fat content and unsaturation, on rodents fed with a high-fat diet (HFD). HFD is a significant source of fatty acids taken up by the liver [17]. We identified the specific lipid species that are altered in liver of HFD fed animals due to insulin resistance and fatty liver conditions predisposed to diabetes.

## Materials and Methods

### Animals and Animal Diet

All animal experiments were approved by the institutional animal care and use committee of the biological resource center, A*STAR, Singapore. Male F344 rats (CLEA Japan, Inc. Tokyo, Japan) were received at 4 weeks of age, placed in individual cages, and randomly assigned to chow diet (n = 8) and HFD groups (n = 8). The animals were fed with their respective diets from the 5^th^ week until the completion of the study (24^th^ week). The FA composition of the HFD (Research Diets, New Brunswick, New Jersey, USA) was 62.4% saturated fat, 30.7% monounsaturated fat, 6.9% polyunsaturated fat.

### Biochemical Measurements

We measured plasma triglyceride, glucose, cholesterol and insulin levels at 12, 18 and 24 weeks of age. All the animals were fasted for 13 hours before blood collection. Bleeding was performed from the lateral tail vein using a rodent restrainer. Plasma glucose, cholesterol and triglyceride were evaluated by enzymatic colorimetric method using hexokinase, cholesterol esterase, cholesterol oxidase and fossati steps respectively (Quest Lab Pvt. Ltd Singapore). Plasma insulin was measured by using an ultra-sensitive ELISA kit (Crystal Chem Inc., Illinois, USA). An oral glucose tolerance test (OGTT) was performed at week 18 after an overnight fasting. Glucose (2 g kg^−1^) was administered to rats by oral gavage injection and blood samples were collected from the tail vein at 0, 10, 30, 60 and 120 min. The total area under the glucose curve was determined from time 0 to 120 min (AUC 0–120 min) after glucose administration as described in our earlier work [18].

### *In vivo* MR Imaging and Spectroscopy

All animals were subjected to magnetic resonance imaging (MRI) and localized MRS. Prior to *in vivo* experiments, animals were initially anesthetized with 3% isofluorane in a dedicated chamber. During the course of MRS experiments, isofluorane levels were reduced to 1.5–2.0% in combination with medical air and medical oxygen. *In vivo* imaging and spectroscopy were performed using a 7 T Bruker ClinScan (Siemens VB15) MRI/MRS scanner equipped with a 72 mm volume resonator for RF transmit and 20 mm receive only surface coil. Rats were positioned prone on the surface coil with the liver on top of the coil. The respiratory rate and body temperature were monitored using physiological monitoring system (ML880 16/30 power lab system, AD Instruments, Spechbach, Germany). The temperature probe was placed in the rectum of the rat and body temperature was maintained at 37°C by circulating hot water through a rat cradle on which the animal was resting. *In vivo* measurements were performed on animals in both the chow diet group and HFD group at 12, 18 and 24 weeks of age. Volume localized point resolved spectroscopy sequence (PRESS) with water suppression experiments were performed on a 4×4×4 mm^3^ voxel within the liver using TR = 4000 ms, TE = 13 ms, 128 averages, and 2048 complex points with a spectral width of 3500 Hz. A respiratory triggered gating module was incorporated into the PRESS sequence with a trigger delay of 20 ms and an animal breathing stabilized at 60–65 cycles per minute. Water unsuppressed spectra were acquired under identical conditions with similar parameters but only 4 averages. Lipid estimates were corrected for *T*_2_ relaxation. Fat content was estimated by the ratio of n-methylene signal (1.30 ppm) to the sum of n-methylene and water signals. An unsaturation index was estimated using the ratio of olefinic signal (5.30 ppm) to the sum of olefinic, methylenes (1.30 ppm and 2.06 ppm) and methyl signals (0.90 ppm) [19].

### Liquid Chromatography Coupled with Mass Spectrometry (LC-MS)

Animals were sacrificed at the 24^th^ week just after the final *in vivo* MR experiments. The liver tissues were snap frozen in liquid nitrogen. Lipids were extracted and quantified using methodology as described in our earlier work [20], [21], [22], [23]. About 20–30 mg of liver tissue was homogenized in 900 µL of chloroform/methanol, 1/2, v/v (Merck Pte. Ltd., Singapore) and incubated on a vacuum chamber in a dark room for 1 h with agitation. After incubation, 0.3 mL of chloroform was added to the homogenate, followed by 0.4 mL of ice-cold water. The homogenate was then vortexed for 30 s followed by centrifugation for 2 min at 9000 rpm. The bottom organic phase was carefully transferred to an empty tube and 0.5 mL of ice-cold chloroform was added and re-extracted to collect the residual lipids fractions. The two organic extracts were then combined and dried under nitrogen. Individual classes of polar lipids were separated using an Agilent 1200 HPLC system before introduction into a 3200 Q-Trap mass spectrometer (Applied Biosystems) with HPLC conditions: Luna 3-mm silica column (i.d. 150×2.0 mm), mobile phase A (chloroform∶methanol∶ammonium hydroxide, 89.5∶10∶0.5), mobile phase B (chloroform∶methanol∶ammonium hydroxide∶water, 55∶39∶0.5∶5.5); flow rate 300 µL min^−1^; 5% B for 3 min, then linear increase of B up to 30% in 24 min, follow by 5 min under these conditions, and then linear change to 70% B in 5 min. Mass spectrometry was recorded under both positive and negative electron-spray ionization (ESI) modes depending on the fraction [21] (conditions: Turbo Spray source voltage, 5000 and - 4500 V for positive and negative modes, respectively) with EMS scan type (conditions: source temperature, 300°C; GS_1_: 40.00, GS_2_: 40.00, curtain gas: 25.). Various lipid fractions including phosphatidylcholine (PC), sphingomyelin (SM), ceramide (Cer) and Glucocyl-ceramide (GluCer) were acquired in the positive ESI mode while phosphatidylethanolamine (PE), phosphatidylinositol (PI), phosphatidylserine (PS), phosphatidylglycerol (PG), phosphatidic acids (PA) and gangliosides mannoside 3 (GM3) were measured in the negative ESI mode [21]. Individual lipid species were quantified by comparison with spiked internal standards PC-14∶0/14∶0, PE-14∶0-14∶0, PS- 14∶0/14∶0, PA-17∶0/17∶0, PG-14∶0/14∶0, d31-PI18∶1/16∶0, C17-LPC, C17-LPA, C17-LPS, C17-Cer, C8-GluCer, C12-SM and C17-ganglioside GM3 obtained from Avanti polar lipids (Alabaster, AL, USA). The molar fractions of individual lipid species and each lipid class were normalized by summation of all polar lipid species. TGs were separated from polar lipids on an Agilent Zorbax Eclipse XDB-C18 column (i.d. 150×4.66 mm), with chloroform∶methanol∶0.1 M ammonium acetate (100∶100∶4) as mobile phase at a flow rate of 0.25 mL min^−1^. TGs were analyzed by using a modified version of reversed phase HPLC/ESI/MS with d5-TG 48∶0 (CDN isotopes) as internal standard [22]. Cholesterol esters were analyzed with corresponding d6-C18 cholesterol ester (CDN isotopes) as internal standards [23]. The unsaturation index from the LC-MS data were computed by the ratio of the absolute concentration of all unsaturated lipids to the concentration of saturated lipids. Triglycerides with no double bonds were defined as saturated, with 1 to 3 unsaturations as mono-unsaturated (considering up to one double bond for each fatty acyl chain) and more than 3 double bonds as poly-unsaturated. Two unsaturation indices were derived considering either all unsaturated fatty acids (mono- and poly-unsaturated, n≥1) or only the poly-unsaturated ones (n>3).

### mRNA Analysis

Total RNA was extracted from the liver samples using trizol reagent (Invitrogen) and treated with DNase I prior to cDNA conversion using the revertAid H minus first strand cDNA synthesis kit (Fermentas, USA) with oligo d(T) 18 primer according to manufacturer's instructions. For real time qPCR, cDNA samples were analyzed in triplicates using the SYBR Green PCR Master Mix reagent kit (Applied Biosystems) on a StepOnePlus Real-Time PCR System (Applied Biosystems). Relative mRNA levels were calculated and normalized to glyceraldehyde 3-phosphate dehydrogenase GAPDH (CAAGGTCATCCATGACAACTTTG) and (GGCCATCCACAGTCTTCTGA), used as an endogenous control gene. The primer sequences used were as follows: peroxisome-proliferator-activated receptor α; PPARα (TGTCATCACAGACACCCTCTCTC) and (TCATCTGTACTGGTGGGGACA), sterol regulatory element binding factor; SREBF1 (CTGCTTTGGAACCTCGTCCG) and (GCCTCCTGTGTACTTGCCCAT), stearoyl-CoA desaturase 1; SCD1 (CCTACGACAAGAACATTCAATCTC) and (TTGATGTGCCAGCGGTACTCACTG). Fatty acid translocase; CD36 (Rn02115479_g1), mitochondrial uncoupling protein 2; UCP2 (Rn01754856_m1) (Taqmann, Life Technologies, CA, USA).

### Histology

Rat liver sections were stained for Oil Red O and hematoxylin & eosin (H & E) to assess the fat accumulation and hepatocyte inflammation, respectively. After the terminal study the livers were excised and fixed in 10% formalin for 24 h and embedded in paraffin wax after dehydration. Formalin fixed rat livers were sectioned at 8 µm and slides were rinsed with PBS (pH 7.4). After passing dry air, the slides were placed in 100% propylene glycol for 2 min, and stained in 0.5% Oil Red O solution in propylene glycol for 30 min. The slides were transferred to a 85% propylene glycol solution for 1 min, rinsed in distilled water for 2 times and processed for hematoxylin counter staining. The steatosis/steatohepatitis was evaluated using a semi-quantitative scoring system [24]. The scoring was based on a chow diet group of animals for which if liver acini did not show lipid vacuoles, a score of zero was given (baseline). Acini having lipid vacuoles up to 33% (mainly macrovesicular type) were considered as score 1. Acini with 34–66% of lipid vacuoles were scored 2 while acini having over 66% of lipid vacuoles were ranked 3.

### Data Analysis and Statistics

Spectroscopic data were processed and analyzed using the LCmodel software [25]. Lipid concentrations for both chow diet and HFD groups were estimated by fitting the resonances of methyl, n-methylene, allylic methylene, and the unsuppressed water signal. The results are expressed as the mean ± s.e.m. (standard error of the mean). Statistical analysis was performed by MedCalc, with significant differences between means identified using paired two-tailed t-test. Differences were considered significant at *P*<0.05.

Unsupervised multivariate factor analysis was performed using principal component analysis (PCA) (Unscrambler 10.2 software) on all quantitative data measured through LC-MS techniques. The PCA results are represented in terms of scores and correlation loadings plots. These scores and correlation loading vectors provide a concise and simplified description of the variance hidden in the dataset [26], [27]. In the current PCA model the term ‘explained validation variance’ (EVV), expressed in percentage, is defined as the proportion of the variance in the data explained by the model.

## Results

### Bodyweight and Biochemical Measurements

**Figure 1:**
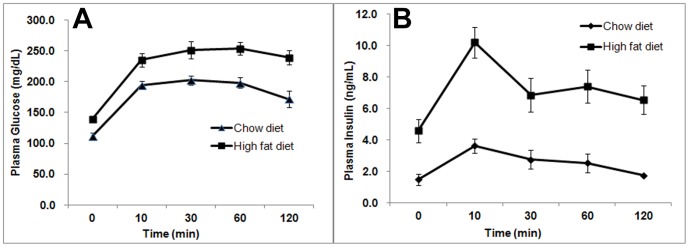
OGTT measurements. Time course of oral glucose tolerance test measurements from (A) plasma glucose and (B) plasma insulin for chow diet and HFD fed rats.

HFD fed animals gained significant body weight during the 9^th^ and 10^th^ weeks. The average body weight of HFD group at the 12^th^, 18^th^ and 24^th^ week was significantly (*P*<0.001) higher than chow diet group (Table 1). The relative increase in percentage body weight of HFD rats at 12, 18 and 24 weeks were 20%, 35% and 42% higher than the chow diet rats respectively. Blood plasma glucose, insulin, total cholesterol and TG measured in HFD and chow diet groups of rats are listed in Table 1. The total TGs (*P*≤0.002) and cholesterol content (*P*≤0.001) of HFD animals were significantly higher than the chow diet fed animals for all measurements. The plasma glucose and insulin concentrations after OGTT performed at 18 weeks are shown in Figure 1A and B, respectively. Plasma glucose and insulin concentrations calculated by the area under the curve from 0–120 mins were significantly (*P*<0.001) higher in HFD fed rats compared to chow diet fed rats. Plasma glucose and insulin concentration from 0–120 mins for HFD rats was 29108 mg/dL and 876 mg/dL compared to 22607 mg/dL and 296 mg/dL in chow diet fed rats respectively. The plasma glucose and insulin concentrations of HFD rats at the 12^th^, 18^th^ and 24^th^ weeks were significantly higher (*P*<0.001) than chow diet fed rats (Table 1).

**Table 1:** Biochemical and body weight measurements of chow diet and HFD fed rats.

Biochemical measurements	Chow diet animals			High fat diet animals*		
	12^th^ week	18^th^ week	24^th^ week	12^th^ week	18^th^ week	24^th^ week
**Cholesterol (mg/dL)**	46.24	49.06	53.64	119.50	133.37	151.63
**Triglyceride (mg/dL)**	78.54	82.65	85.97	401.74	423.61	469.94
**Glucose (mg/dL)**	102	115	122	136	148	164
**Insulin (ng/mL)**	1.14	1.31	1.40	4.26	4.71	5.21
**Body weight (gm)**	231	274	342	278	371	487

The blood plasma cholesterol, triglycerides, glucose, insulin and body weight were significantly (*P*<0.05).

* higher in HFD fed rats.

### Magnetic Resonance Spectroscopy

**Figure 2:**
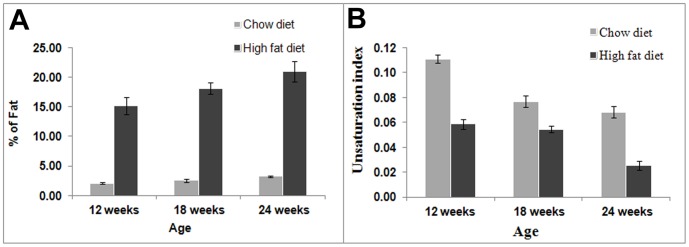
Liver fat and unsaturation indices estimated by *in vivo* MRS. Estimation of (A) liver fat (%) and (B) unsaturation indices by *in vivo* MRS from chow diet and high fat diet fed animals at the 12^th^, 18^th^ and 24^th^ weeks. Liver fat content was significantly (*P*<0.001) higher in HFD fed rats and unsaturation indices were significantly (*P*<0.005) higher in chow diet fed rats for all age groups.

The liver fat was measured longitudinally in chow diet and HFD fed animals at the 12^th^, 18^th^ and 24^th^ weeks of age. Most of the lipid resonances are well resolved at 7 T compared to lower field magnets. The representative *in vivo* spectra obtained from both HFD and chow diet fed rats are shown in Figure S1A and B. The signals from methyl (0.9 ppm), n-methylene (1.30 ppm), allylic methylene (2.06 ppm), α-methylene (2.20 ppm) and olefinic (5.30 ppm) groups are assigned in the spectra. Figure 2A shows the liver fat content of chow diet and HFD fed rats for different age groups. Total liver fat contents of the HFD and chow diet fed animals at 12 weeks were 15.18±1.47% and 2.11±0.12%, respectively. At 18 weeks the fat content in HFD animals increased to 18.14±0.91% compared to the chow diet group 2.55±0.23%. At 24 weeks the liver fat content increased to 20.97±1.71% and 3.29±0.13% for the HFD and chow diet animals, respectively. The liver fat content was significantly (*P*<0.001) higher in HFD animals compared to the chow diet group for all time points in the study. The liver fat of HFD fed animals increased significantly with age from 12 to 18 to 24 weeks. Figure 2B shows the unsaturation indices (UI) of chow diet and HFD animals at different ages. The UI of chow diet fed animals was always higher than the HFD fed group at all ages (*P*<0.005).

### *In vitro* LC-MS Studies

**Figure 3:**
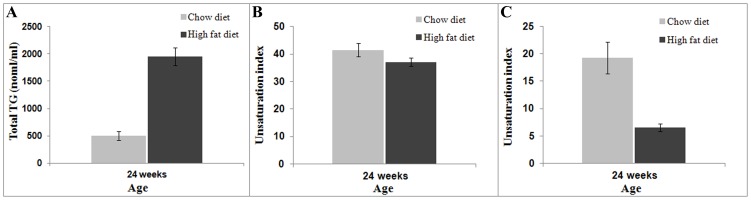
Triglycerides and unsaturation indices estimated from LC-MS. Estimation of (A) total triglycerides (B) unsaturation indices (by considering unsaturated FAs with n≥1 and (C) unsaturation indices (by considering unsaturated FAs with n>3) at 24 weeks in high fat diet and chow diet fed rats.

The liver was harvested after the terminal *in vivo* study at 24 weeks and tissue samples were subjected to LC-MS analysis. The mean TG content in HFD animals (Figure 3A) was 1983±356 nmol/mL while it was significantly lower (511±221 nmol/mL, *P*<0.001) for the chow diet group. Figure 3B and C show the two unsaturation indices of HFD and chow diet groups determined by considering as unsaturated FAs either from n≥1 or from n>3, respectively (both definitions given in the materials and methods section). The unsaturation index for the chow diet fed animals was significantly higher (*P*<0.002) when considering the poly-unsaturated fatty acids for its calculation.

**Figure 4:**
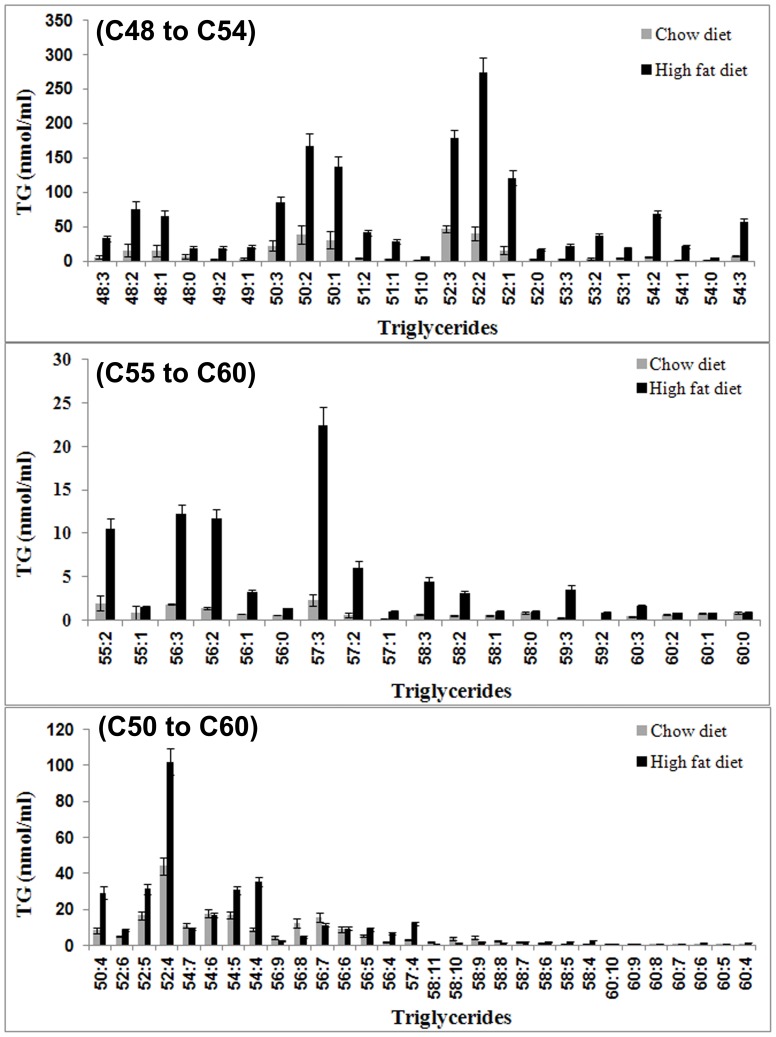
Saturated and unsaturated triglycerides by LC-MS. Concentrations of saturated and unsaturated TGs in HFD and chow diet fed rats estimated by LC-MS at 24 weeks. Triglycerides C50∶2, C50∶1, C52∶3, C52∶2 and C52∶1 were found to be most abundant (>100 nmol/ml) and significantly higher in HFD fed rats compared to chow diet fed rats. Concentrations of other TGs were less than 100 nmol/ml but significantly higher in HFD rats.

Figure 4 shows the saturated and unsaturated TGs in HFD and chow diet groups. Individual concentrations are provided in Table S1. The triglycerides 50∶2, 50∶1, 52∶3, 52∶2 and 52∶1 were found to be the most abundant (120.99 to 273.60 nmol/mL) and significantly higher (*P*<0.05) in HFD rats than in the chow diet fed group. Concentrations of the triglycerides 48∶2, 48∶1, 50∶3, 54∶2, 54∶3 were in the range 56.73 to 85.44 nmol/mL in HFD rats. The rest of the TG concentrations were in the lower range of 0.8 to 33.01 nmol/mL but were still higher in HFD rats compared to chow diet fed rats (*P*<0.05). In chow diet group of animals the concentrations of all the saturated TGs were in the lower range of 0.1 to <50 nmol/ml. The unsaturated TGs including 50∶4, 52∶5, 52∶4, 54∶5, 54∶4, and 57∶4 with concentration in the range 5 to 102 nmol/ml in HFD rats were significantly (*P*<0.05) higher than chow diet fed rats. However, the poly-unsaturated triglycerides 54∶7, 56∶8, 56∶7, 58∶11, 58∶10, 58∶9, 58∶8 and 60∶10 were significantly higher (*P*<0.05) in chow diet fed rats than in HFD rats.

In addition to the TGs, we also estimated the concentration of phospholipids [PC, PE, PI, PG, PS, PA] and sphingolipids [SM, Cer, GluCer, GM3]. Both chow diet and HFD fed groups exhibit a similar concentration for each of the molecule studied (Figure S2A and B), except for lysophospholipids (LPC and LPE, Table 2). Total LPC levels, as well as the LPCs 18∶1, 18∶0 and 20∶0 and LPEs 16∶0, 18∶1 and 18∶0 were significantly (*P*<0.05) higher in HFD rats (Table 2). Cholesterol ester (CE) content evaluated using LC-MS in HFD animals was higher 226±50 nmol/mL than chow diet group 15±2 nmol/ml mL (*P*<0.001).

**Table 2:** Concentrations of LPCs and LPEs in liver tissues of chow diet and HFD fed rats at 24 weeks.

LPC and LPE	Chow diet (µmol/ml)	HFD (µmol/ml)	*P* value
**LPC18∶0**	0.0063±5×10^−4^	0.008±6×10^−4^	0.005
**LPC18∶1**	0.0012±6×10^−5^	0.0023±2×10^−4^	<0.005
**LPC20∶0**	0.0027±2×10^−4^	0.004±5×10^−4^	<0.04
**LPE16∶0**	0.0147±1×10^−3^	0.0184±7×10^−4^	<0.005
**LPE18∶1**	0.0023±9×10^−5^	0.0048±5×10^−4^	<0.001
**LPE18∶0**	0.010±6×10^−4^	0.017±9×10^−4^	0.001

Concentrations of lysophosphatidylcholines (LPC) and lysophosphatidylethanolamines (LPE) were significantly higher in liver tissues of HFD fed rats.

The PCA was performed on LC-MS data. The model optimally fitted the data with 96% and 3% of the total variance explained on PC1 and PC2, respectively. The PCA score plot (figure not shown) of the quantitative LC-MS data showed well delineated clusters of HFD and chow diet fed rats in the multidimensional space. The loading plot (Figure S3) highlighted those key metabolites which were predominantly accounted for variability along PC1 and PC2 vectors. The PCA isolated the unsaturated TGs 52∶2, 52∶3, 52∶1, 54∶2, 50∶2, 50∶1, 48∶2, 48∶1, 50∶3, 54∶3 on PC1 axis and were present in higher concentrations.

### mRNA Analysis

**Figure 5:**
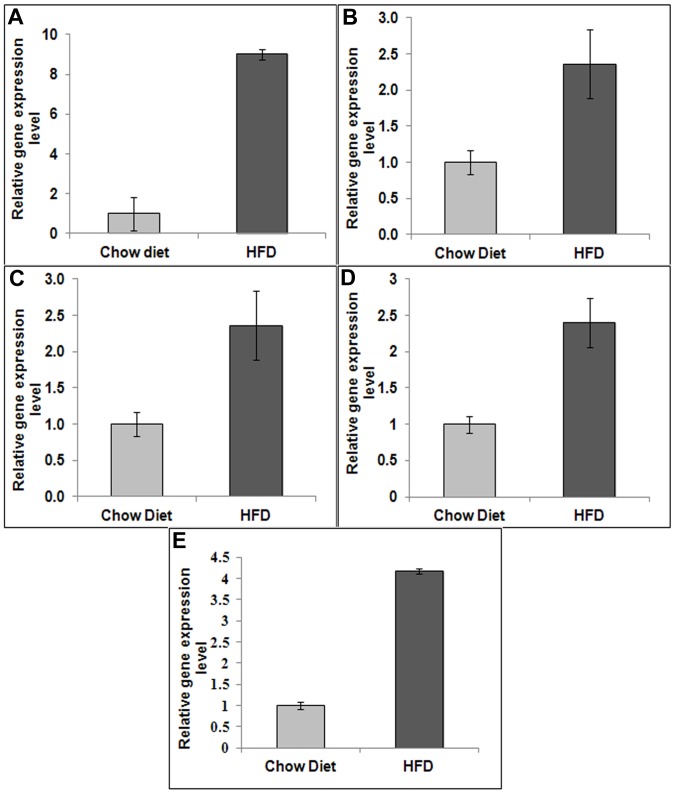
mRNA expression analysis. mRNA expression of (A) CD36, (B) SREBF1, (C) PPARα, (D) SCD1, and (E) UCP2. The expression of all these genes were significantly higher for HFD rats than for chow diet fed animals.

The real-time PCR mRNA expression analysis of various genes for both chow diet and HFD rats are shown in Figure 5. We found an increased expression (*P*<0.05) of mRNA levels of fatty acid translocase (CD36: 9 fold), peroxisome-proliferator-activated receptor α (PPARα: 2.35 fold), sterol regulatory element binding factor (SREBF1: 2.40 fold), stearoyl-CoA desaturase 1 (SCD1: 2.20 fold), mitochondrial uncoupling protein 2 (UCP2: 4.17 fold) in the HFD animal livers.

### Histopathologic Assessment of Hepatic Steatosis

**Figure 6:**
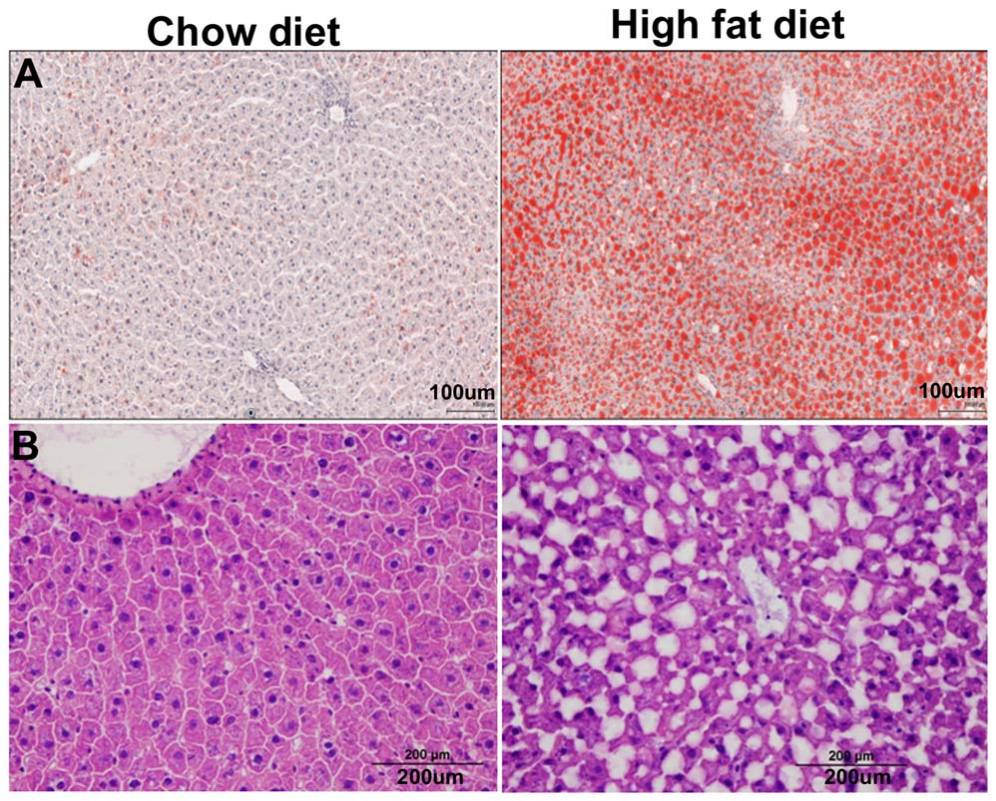
Liver histology. (A) Oil red O staining and (B) Hematoxylin-eosin staining of HFD and chow diet fed rat liver tissues at 24 weeks. The HFD fed rats showed histopathological features of hepatic steatosis with a score of 3 indicating over 68% of acini occupied by lipid vacuoles compared to chow diet fed rats.

Representative H & E and Oil Red O stained sections of 24 weeks old rat liver from HFD and chow diet animals are showed in Figure 6A and B, respectively. The fat deposition (lipid droplets) in stained sections of the HFD fed animals is of mixed type including macrovesicular type where in either single large fat droplet displacing the nucleus or the presence of smaller well-defined intracytoplasmic droplets. Based on the scoring method [24], the HFD fed animals showed histopatholgical features of hepatic steatosis with a score of 3 indicating over 68% of acini were occupied by lipid vacuoles compared to chow diet fed animals. Accumulation of lipid vacuoles was of both macro- and micro-vesicular patterns. There were clear, well delineated and the cytoplasmic vacuoles in the hepatocytes were of variable sizes with mainly midzonal and paracentral distributions. There were 68±1.08% lipid vacuoles in the liver of HFD rats compared to 4.59±1.54% only for the chow diet group. HFD animals displayed histopatholgical features of hepatic steatosis.

## Discussion

Dietary intake of high amounts of saturated FAs and low amounts of polyunsaturated fatty acids can cause NAFLD [28]. In this study, we investigated the longitudinal changes of lipid composition in a high saturated fat diet fed rodent model using *in vivo* MRS and LC-MS techniques.

Our HFD model showed significant weight gain starting at the 9^th^ week. The blood plasma analysis of the HFD animals showed hyperinsulinemia and hypertriglyceridemia confirming insulin-resistant conditions [29], [30]. High saturated fat diet intervention resulted in an increase of body weight, increase in triglycerides and cholesterol which lead to the metabolic consequence of insulin resistance from the 12^th^ week. The liver fat of HFD groups as assessed by MRS, was higher by 13–17% compared to the respective chow diet groups. This was confirmed by measuring the liver TG content by LC-MS and also on histology. Histology results from HFD fed rats showed excessive accumulation of triglycerides with 68% of lipid vacuoles occupying the hepatocytes at 24 weeks. The 9-fold increase in CD36 mRNA expression in HFD rat livers suggested enhanced fatty acid transport in the fatty liver resulting in an increased demand for the oxidation of fatty acids. Hepatic PPARα associated with the attenuation of insulin signaling and hepatic steatosis was upregulated by 2.35 fold in HFD rats. The SREBF1 was elevated 2.4 fold in HFD fed rats implicating the cause of insulin resistance and hepatosteatosis as confirmed by histology. Hepatic mitochondrial oxidant production is one of the primary mechanisms that promotes oxidative stress during NAFLD, NASH and type 2 diabetes conditions [31], [32]. UCP2 is mitochondrial inner membrane protein present in variety of tissues including liver (hepatocytes) and its main function involves the control of mitochondria-derived oxidant production [33], [34], [35]. Studies on different tissues of animal models confirmed that the baseline expression of UCP2 gene in hepatocytes is undetectable but it shows significant up-regulation in the liver during pathologic conditions associated with steatosis [34]. The 4-fold increased expression of UCP2 levels in HFD group supported the involvement of UCP2 gene in the pathogenesis of NAFLD.

In addition, we showed that the FA composition of the liver is altered by HFD feeding. The unsaturation indices as determined by *in vivo* MRS at 12, 18 and 24 weeks and by LC-MS at 24 weeks were significantly lower in HFD rats. The UIs estimated by MRS using resonances of olefinic, methyl, methylene and allylic methylene at 24 weeks showed a 2.6 fold increase in chow diet fed rats. The UIs estimated by LC MS were 1.12 and 2.9 fold higher in chow diet fed rats when considering both mono- and poly-unsaturated FA and only polyunsaturated fatty acids, respectively. This 2.9 fold higher UI obtained by calculating only polyunsaturated fatty acids is comparable with the MRS result (2.6 fold). The *in vivo* MRS and *in vitro* LC-MS techniques provided complimentary information and had a good agreement for the estimation of unsaturation in chow diet and HFD fed groups. Both methods demonstrated decreased unsaturation in HFD rats compared to chow diet rats. The accuracy of estimating the unsaturation by 1D MR methods can be improved by including bi-allylic methylene (2.8 ppm) signal which is not well resolved. Localized 2D L-COSY technique [36] provides improved spectral resolution where *J* coupled multiplet resonances are dispersed over two spectral dimensions. The L-COSY techniques has been utilized to estimate unsaturation using the cross peaks generated by the scalar couplings between olefinic and allylic, diallylic methylene protons [37] in skeletal muscle. This technology can be further developed for liver applications with appropriate motion compensation and optimization of acquisition time within a clinical setting.

The decrease in unsaturation in the HFD group might be due to alteration in fatty acid composition because of changes in desaturase expression or activities. Donelly et al. have shown that dietary fatty acids contribute to the fatty acid pool in the liver [17]. As such, the high proportion of saturated fat and decreased availability of polyunsaturated lipid components in the HFD contributed to the low unsaturation index. Degree of unsaturation can be influenced by the activity of desaturases. SCD1 catalyzes the conversion of the saturated fatty acyl-CoAs, to their respective monounsaturated fatty acyl CoAs. The SCD1 was up regulated in the livers of HFD mice by 2.2 fold. SCD1 up regulation contributed to increase in monounsaturated fatty acids (Table S1). In spite of increased mono-unsaturation, the proportion of the available saturated fatty acids was still abundant in the HFD group resulting in lower unsaturation index. The upregulation of SCD1 in HFD rats confirmed its crucial role in the pathogenesis of diet-induced hepatic insulin resistance [38]. It was established that rate-limiting nature of desaturation enzymes contribute to the development of the diabetic condition [39]. A reduced degree of unsaturation was reported in the skeletal muscle of overweight and obese subjects [40]. Earlier MRS studies showed the changes in degree of unsaturation in skeletal muscle of normal, overweight and obese human subjects [16] and bone marrow composition in osteoporosis subjects [19]. Relatively high levels of saturated fatty acids and low levels of polyunsaturated fatty acids are found in individuals with insulin resistance and metabolic syndrome [41]. Our study suggested that the same phenomenon was observed in the liver of rats fed with a HFD and may also be relevant to the pathogenesis of NAFLD and insulin resistance associated with high fat feeding.

We also noticed a decreasing trend of FA unsaturation in chow diet fed rats over the age which might be due to the redistribution of the fatty acid composition. It was shown that ageing can alter liver mitochondrial membranes influencing the free radical production and reduce unsaturation [42]. During ageing there is a redistribution between types of unsaturated fatty acids resulting in transition from highly unsaturated fatty acids to less unsaturated fatty acids [43]. In addition, it was found that impairment in activity of delta-6-desaturase (D6D) due to ageing process results in decreased synthesis of n-6 and n-3 polyunsaturated fatty acids which is also a contributing factor in reducing the unsaturation index over time [44].

The multivariate analysis showed the triglycerides (52∶2, 52∶3, 52∶1, 54∶2, 50∶2, 50∶1, 48∶2, 48∶0, 48∶1, 50∶3, 54∶3) accounted for increased fat in the liver. Rhee et al. [45] showed that triglycerides 48∶0, 48∶1 and 52∶1 in human plasma of diabetic individuals are associated with increased risk of diabetes mellitus. Our current results suggests that the accumulation of these fatty acids in liver tissues of HFD fed rats may also be important in increasing the risk of diabetes mellitus. Cholesterol ester hydrolase (CEH) plays a vital role in hepatic cholesterol homeostasis and its activity is proportional to production of cholesterol ester. Three fold higher CEH activity was reported in patients with acute hepatitis compared to normal livers [46], [47]. In the present study the excessive accumulation of cholesterol ester in HFD rats confirmed the steatosis condition of the liver indicating the risk of hepatitis. Increased incorporation of saturated fat and cholesterol into the cell membranes increase the membrane rigidity thereby reducing the number of insulin receptors and their affinity to insulin, causing an insulin resistance in the local tissue. This increased availability of hepatic saturated fatty acids in HFD fed animals reduces hepatic fatty acid oxidation and triglyceride export. This in turn would increase hepatic fatty acid and triglyceride synthesis which would augment the triglyceride accumulation in the liver of HFD fed animals. LPC is an important signaling molecule with diverse biological functions and involved in regulating cellular inflammation [48], [49]. Plasma, liver and skeletal muscle LPC levels are increased in the obese diabetic db/db mouse and these findings support that LPC may be involved in mediating insulin resistance in obesity [50]. Increased abundance of LPCs was shown to induce hepatocellular death caused by mitochondrial membrane depolarization [51], [52].

The HFD fed rat livers showed increased concentration of lysophospholipids (LPC and LPE). In particular, excessive accumulation of LPC 18∶1, 18∶0, 20∶0 and LPE 16∶0, 18∶0, 18∶1 in HFD fed rats might be due to liver inflammation indicating the increased risk of diabetes [53]. Production of these lysophospholipids *in vivo* is usually mediated by the release and/or activation of the enzyme phospholipase A2 (PLA2) under inflammatory conditions. PLA2 is present in neutrophilic granulocytes and its activation under inflammatory condition is also associated with generation of reactive oxygen species. In a recent clinical study, the LPE and LPCs were significantly higher in inflammatory livers compared to healthy liver as confirmed by ^31^P MRS [54].

In conclusion, HFD increased the total fat fraction, and altered the fatty acid composition which might be due to the composition of the diet, or alterations in desaturase enzymes. The change in unsaturation is relevant to the pathogenesis of NAFLD and insulin resistance. Perturbations in lysophospholipids (LPC and LPE) might provide early information on oxidative stress/inflammation in NAFLD before the incidence of diabetes. These markers may be useful to study the link between a HFD and medical conditions in humans. Further studies are required to explore the possibilities of using unsaturation index in scaling the severity of the NAFLD in clinical practice. These studies could be extrapolated to evaluate the metabolic response during interventions including exercise and drugs.

## Supporting Information

Figure S1 ***In vivo*** **liver spectra from HFD and chow diet fed rats.** Representative *in-vivo* liver spectra from (A) HFD and (B) chow diet fed rats. The signals from methyl (0.9 ppm), n-methylene (1.30 ppm), allylic methylene (2.06 ppm), α-methylene (2.20 ppm) and olefinic (5.3 ppm) groups are assigned in the spectra.
(TIF)

Figure S2 **Concentration of phospholipids and sphingholipids from HFD and chow diet fed rats.** A. Concentrations of sphingomyelin (SM), ceramide (Cer), phosphatidylcholine (PC), phosphatidylethanolamine (PE), phosphatidylinositol (PI), phosphatidylserine (PS). B. Glucocyl-ceramide (GluCer), phosphatidic acids (PA), phosphatidylglycerol (PG), gangliosides mannoside 3(GM3) in HFD and chow diet fed rats.
(TIF)

Figure S3 **Multivariate analysis of lipid components.** Multivariate analysis of lipid components in HFD and chow diet fed rats. Loading plot highlighted the key TGs contributing to the maximum variance between the two groups.
(TIF)

Table S1 **Concentrations of saturated and unsaturated triglycerides.** Concentrations of saturated and unsaturated triglycerides in liver of chow diet and HFD fed rats at 24 weeks
(DOCX)
