# Supplementary figures and images for: Evaluation of Dietary Effects on Hepatic Lipids in High Fat and Placebo Diet Fed Rats by In Vivo MRS and LC-MS Techniques
Source: PLoS One. 2014 Mar 17;9(3):e91436. doi: 10.1371/journal.pone.0091436 (PMC3956606; doi:10.1371/journal.pone.0091436)

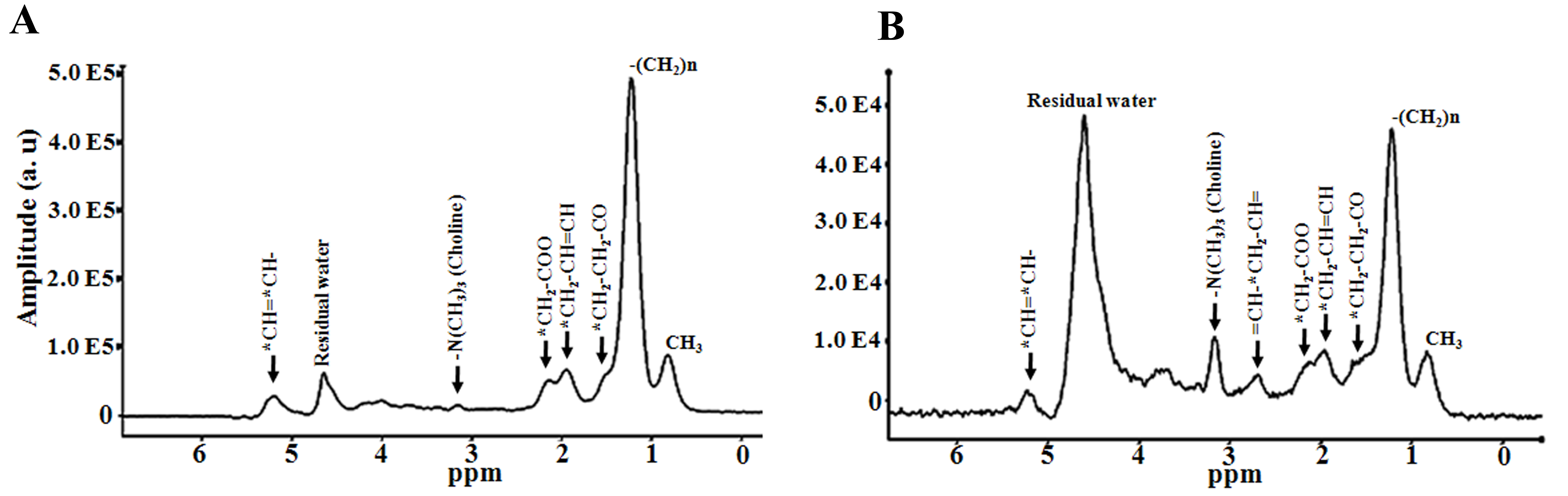

Supplement: Figure S1 — In vivo liver spectra from HFD and chow diet fed rats. Representative in-vivo liver spectra from (A) HFD and (B) chow diet fed rats. The signals from methyl (0.9 ppm), n-methylene (1.30 ppm), allylic methylene (2.06 ppm), α-methylene (2.20 ppm) and olefinic (5.3 ppm) groups are assigned in the spectra. (TIF) [file pone.0091436.s001.tif]

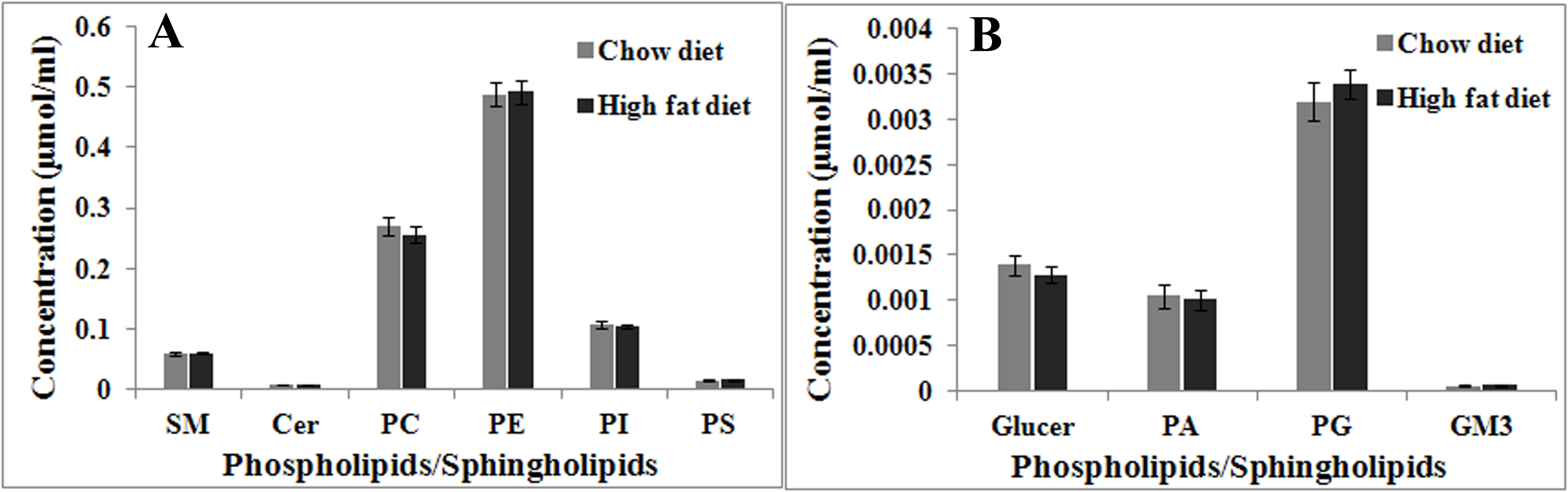

Supplement: Figure S2 — Concentration of phospholipids and sphingholipids from HFD and chow diet fed rats. A. Concentrations of sphingomyelin (SM), ceramide (Cer), phosphatidylcholine (PC), phosphatidylethanolamine (PE), phosphatidylinositol (PI), phosphatidylserine (PS). B. Glucocyl-ceramide (GluCer), phosphatidic acids (PA), phosphatidylglycerol (PG), gangliosides mannoside 3(GM3) in HFD and chow diet fed rats. (TIF) [file pone.0091436.s002.tif]

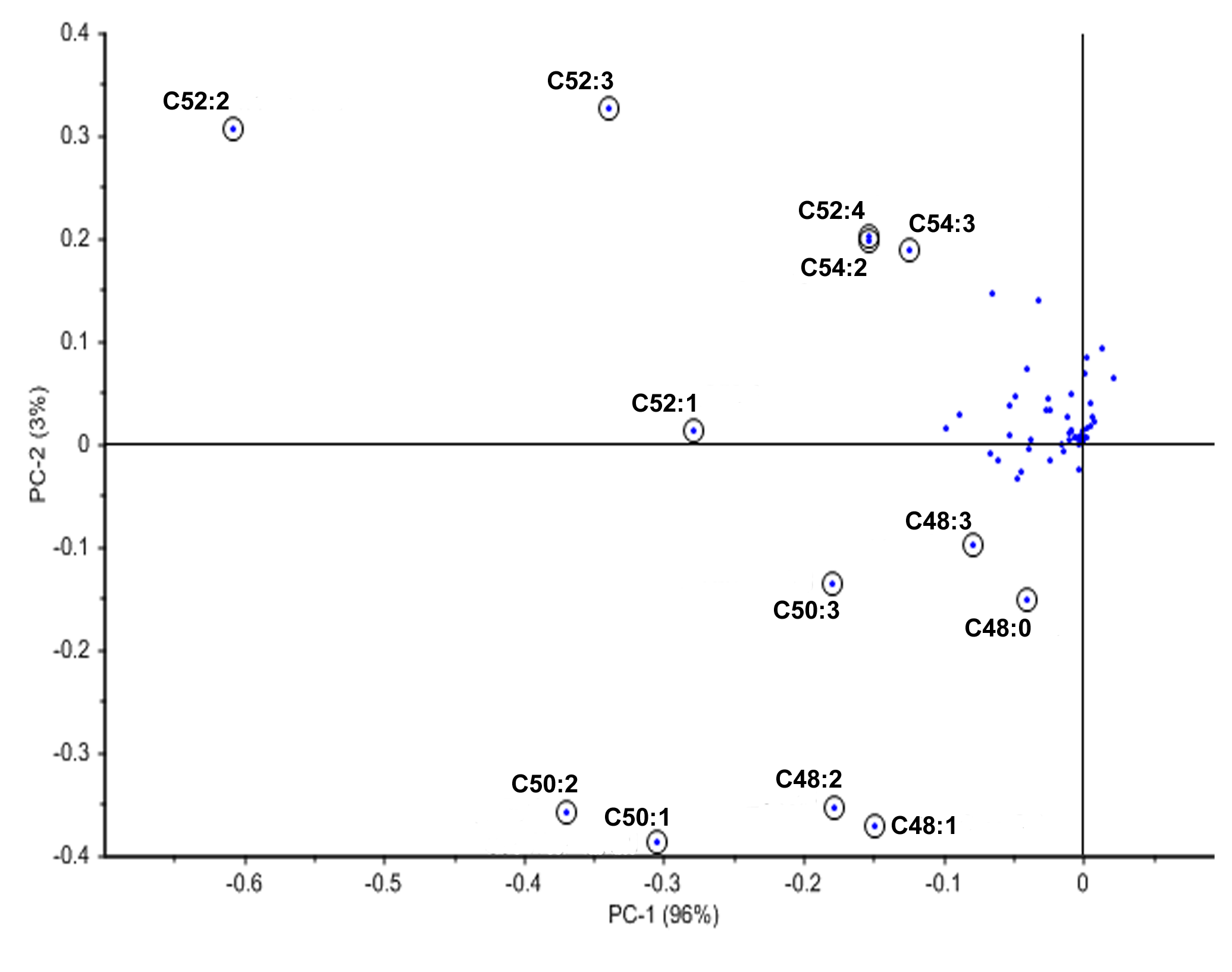

Supplement: Figure S3 — Multivariate analysis of lipid components. Multivariate analysis of lipid components in HFD and chow diet fed rats. Loading plot highlighted the key TGs contributing to the maximum variance between the two groups. (TIF) [file pone.0091436.s003.tif]
