# Supplementary material for: Evaluation of Dietary Effects on Hepatic Lipids in High Fat and Placebo Diet Fed Rats by In Vivo MRS and LC-MS Techniques
Source: PLoS One. 2014 Mar 17;9(3):e91436. doi: 10.1371/journal.pone.0091436 (PMC3956606; doi:10.1371/journal.pone.0091436)
Supplement: Table S1 — Concentrations of saturated and unsaturated triglycerides. Concentrations of saturated and unsaturated triglycerides in liver of chow diet and HFD fed rats at 24 weeks (DOCX) [file pone.0091436.s004.docx]

**Table S1**

**Concentrations of saturated and unsaturated triglycerides in liver at 24 weeks.**

| **Saturated triglycerides** | **Chow diet (nmol/ml ± s.e.m)** | **HFD (nmol/ml ± s.e.m)** |
| --- | --- | --- |
| **48:3** | 6.228 ± 2.785 | 33.012 ± 4.872 |
| **48:2** | 15.873 ± 8.854 | 75.766 ± 10.555 |
| **48:1** | 15.222 ± 8.713 | 65.245 ± 8.908 |
| **48:0** | 6.322 ± 3.329 | 19.170 ± 2.679 |
| **49:2** | 2.351 ± 0.865 | 19.172 ± 2.121 |
| **49:1** | 3.484 ± 1.180 | 21.036 ± 2.138 |
| **50:4** | 8.251 ± 1.850 | 29.231 ± 3.598 |
| **50:3** | 22.848 ± 7.084 | 85.449 ± 8.900 |
| **50:2** | 38.225 ± 13.560 | 167.418 ± 18.712 |
| **50:1** | 30.991 ± 13.026 | 137.944 ± 14.705 |
| **51:2** | 4.183 ± 1.264 | 41.477 ± 4.172 |
| **51:1** | 3.020 ± 1.158 | 28.758 ± 2.566 |
| **51:0** | 1.388 ± 0.327 | 6.773 ± 0.561 |
| **52:6** | 5.034 ± 0.308 | 8.378 ± 0.614 |
| **52:5** | 16.666 ± 2.233 | 31.335 ± 2.871 |
| **52:4** | 44.109 ± 4.678 | 102.044 ± 7.135 |
| **52:3** | 47.142 ± 5.07 | 178.336 ± 12.905 |
| **52:2** | 40.413 ± 9.413 | 273.608 ± 21.651 |
| **52:1** | 16.066 ± 6.211 | 120.999 ± 10.499 |
| **52:0** | 2.948 ± 0.899 | 17.671 ± 1.493 |
| **53:3** | 3.238 ± 0.910 | 22.738 ± 2.489 |
| **53:2** | 3.822 ± 1.343 | 37.624 ± 3.291 |
| **53:1** | 4.851 ± 0.518 | 19.138 ± 1.598 |
| **54:7** | 11.284 ± 1.298 | 9.108 ± 0.712 |
| **54:5** | 16.889 ± 1.998 | 30.745 ± 2.161 |
| **54:4** | 8.856 ± 1.000 | 35.489 ± 2.677 |
| **54:2** | 7.005 ± 0.829 | 68.173 ± 5.158 |
| **54:1** | 2.278 ± 0.253 | 21.272 ± 1.808 |
| **54:0** | 1.447 ± 0.085 | 4.836 ± 0.402 |
| **54:3** | 7.513 ± 0.615 | 56.731 ± 4.961 |
| **55:2** | 1.966 ± 0.864 | 10.566 ± 1.116 |
| **55:1** | 0.874 ± 0.776 | 1.505 ± 0.136 |
| **56:9** | 4.450 ± 0.807 | 2.572 ± 0.253 |
| **56:8** | 12.610 ± 2.503 | 4.644 ± 0.552 |
| **56:6** | 8.969 ± 1.400 | 9.314 ± 1.011 |
| **56:5** | 5.196 ± 0.725 | 9.002 ± 0.781 |
| **56:4** | 2.022 ± 0.186 | 6.781 ± 0.513 |
| **56:2** | 1.442 ± 0.126 | 11.700 ± 1.119 |
| **56:1** | 0.757 ± 0.033 | 3.199 ± 0.317 |
| **56:0** | 0.663 ± 0.023 | 1.382 ± 0.099 |
| **57:4** | 3.044 ± 0.355 | 12.100 ± 1.213 |
| **57:3** | 2.292 ± 0.662 | 22.398 ± 2.145 |
| **57:2** | 0.586 ± 0.255 | 6.022 ± 0.805 |
| **57:1** | 0.125 ± 0.040 | 1.060 ± 0.141 |
| **58:11** | 1.619 ± 0.347 | 0.513 ± 0.050 |
| **58:10** | 4.081 ± 0.958 | 1.201 ± 0.145 |
| **58:9** | 4.372 ± 0.980 | 1.927 ± 0.232 |
| **58:8** | 2.397 ± 0.480 | 1.615 ± 0.151 |
| **58:6** | 1.237 ± 0.146 | 1.899 ± 0.122 |
| **58:5** | 0.748 ± 0.048 | 2.023 ± 0.106 |
| **58:4** | 0.716 ± 0.051 | 2.806 ± 0.263 |
| **58:2** | 0.575 ± 0.059 | 3.083 ± 0.343 |
| **58:1** | 0.568 ± 0.046 | 1.020 ± 0.093 |
| **59:3** | 0.295 ± 0.033 | 3.553 ± 0.487 |
| **59:2** | 0.105 ± 0.027 | 0.906 ± 0.091 |
| **60:10** | 0.948 ± 0.189 | 0.601 ± 0.070 |
| **60:8** | 0.501 ± 0.050 | 0.915 ± 0.074 |
| **60:7** | 0.405 ± 0.029 | 0.759 ± 0.030 |
| **60:6** | 0.428 ± 0.024 | 1.155 ± 0.064 |
| **60:5** | 0.365 ± 0.032 | 1.134 ± 0.070 |
| **60:4** | 0.379 ± 0.045 | 1.347 ± 0.098 |
| **60:3** | 0.429 ± 0.033 | 1.632 ± 0.174 |
| **60:2** | 0.670 ± 0.102 | 0.859 ± 0.091 |

All the TGs were significant (*P* < 0.05) except 60:2, 54:7
